# Inorganic mercury in human astrocytes, oligodendrocytes, corticomotoneurons and the locus ceruleus: implications for multiple sclerosis, neurodegenerative disorders and gliomas

**DOI:** 10.1007/s10534-018-0124-4

**Published:** 2018-06-29

**Authors:** Roger Pamphlett, Stephen Kum Jew

**Affiliations:** 1 Discipline of Pathology, The University of Sydney, Camperdown, Australia; 2 Discipline of Pathology, Brain and Mind Centre, The University of Sydney and Department of Neuropathology, Royal Prince Alfred Hospital, 94 Mallett St, Camperdown, NSW 2050 Australia

**Keywords:** Human brain, Inorganic mercury, Astrocyte, Oligodendrocyte, Corticomotoneuron, Locus ceruleus, Multiple sclerosis, Alzheimer’s disease, Amyotrophic lateral sclerosis (ALS), Brain tumour

## Abstract

Neurotoxic metals have been implicated in the pathogenesis of multiple sclerosis, neurodegenerative disorders and brain tumours but studies of the location of heavy metals in human brains are rare. In a man who injected himself with metallic mercury the cellular location of mercury in his brain was studied after 5 months of continuous exposure to inorganic mercury arising from metallic mercury deposits in his organs. Paraffin sections from the primary motor and sensory cortices and the locus ceruleus in the pons were stained with autometallography to detect inorganic mercury and combined with glial fibrillary acidic protein immunohistochemistry to identify astrocytes. Inorganic mercury was found in grey matter subpial, interlaminar, protoplasmic and varicose astrocytes, white matter fibrous astrocytes, grey but not white matter oligodendrocytes, corticomotoneurons and some locus ceruleus neurons. In summary, inorganic mercury is taken up by five types of human brain astrocytes, as well as by cortical oligodendrocytes, corticomotoneurons and locus ceruleus neurons. Mercury can induce oxidative stress, stimulate autoimmunity and damage DNA, mitochondria and lipid membranes, so its location in these CNS cells suggests it could play a role in the pathogenesis of multiple sclerosis, neurodegenerative conditions such as Alzheimer’s disease and amyotrophic lateral sclerosis, and glial tumours.

## Introduction

Neurotoxic metals have been implicated in the pathogenesis of a number of human nervous system disorders (Caito and Aschner 2015). Clinical, experimental and epidemiological studies suggest that mercury could play a part in the pathogenesis of multiple sclerosis (Aminzadeh and Etminan 2007), Alzheimer’s disease (Mutter et al. 2010) and amyotrophic lateral sclerosis (ALS) (Pamphlett and Kum Jew 2013). A number of pathogenetic mechanisms have been linked to mercury, all of which are suspected to operate in neurodegenerative diseases. These include the production of reactive oxygen species (Lund et al. 1993), apoptosis (Ceccatelli et al. 2010), DNA damage (Crespo-Lopez et al. 2009), RNA damage (Chang 1977), epigenetic changes (Basu et al. 2014) and autoimmunity (Vas and Monestier 2008). However, no convincing link between mercury and neurological disorders has been established. One problem in detecting neurotoxic metals in diseased brains is that the cells that originally contained the metals are likely to have been destroyed by the pathological process by the time the brain is available for examination after autopsy. Furthermore, most studies of mercury in the human nervous system have relied on studying the brains of people years after exposure by which time much of the metal is likely to have been cleared from the brain (Tiffany-Castiglion and Qian 2001).

Inorganic mercury (iHg) appears to be the proximate toxic form of CNS mercury (Charleston et al. 1996) but the location of iHg in the human brain is poorly understood (Clarkson and Magos 2006). We were able to study the cellular location of iHg in a man who had been exposed continuously to mercury for 5 months. Using the histochemical technique of silver nitrate autometallography, iHg had previously been detected in his corticomotoneurons and locus ceruleus neurons, as well as in undefined glial cells in the brain (Pamphlett and Waley 1996). Autometallography can now be combined with immunohistochemistry to detect which classes of cells contain iHg (Pamphlett and Kum Jew 2015) so this combination of methods was used in an attempt to detect more precisely which cells contain iHg in an undamaged human brain that had been exposed to mercury at the time of death.

## Methods

### Clinical details

A 24 year-old man had injected himself intravenously with metallic mercury taken from thermometers (Kedziora and Duflou 1995). X-rays showed collections of mercury in his right ventricle, throughout both lung fields and in the pelvic venous plexuses. He remained asymptomatic but died 5 months later after lacerating his wrists. At autopsy metallic mercury collections were seen on the cut surfaces of his right ventricular myocardium, lungs and pelvic veins.

### Staining inorganic mercury and astrocytes

7 μm sections of formalin-fixed paraffin-embedded blocks taken from the cerebral cortex, which included the primary sensory and motor cortices and underlying white matter, as well as sections from the pons containing the locus ceruleus, were stained with silver nitrate autometallography to detect iHg (Danscher and Stoltenberg 2006). Briefly, sections were placed in physical developer containing gum arabic, citrate buffer, hydroquinone and silver nitrate at 26 °C for 85 min in the dark, then washed in sodium thiosulphate to remove unbound silver, lightly counterstained with hematoxylin and viewed under bright-field microscopy. A positive control section was of mouse spinal cord where spinal motor neurons contained iHg following an intraperitoneal injection of mercuric chloride (Pamphlett and Png 1998). The silver-coated deposits of iHg in cells are seen microscopically as black grains and referred to as AMG^Hg^ (autometallography-demonstrable mercury). To identify iHg in astrocytes AMG^Hg^-stained sections were immunostained with polyclonal rabbit-anti-human glial fibrillary acidic protein (GFAP, DAKO Z0334) at 1:2000 for 60 min at 37 °C and visualised with diaminobenzidine tetrahydrochloride. Oligodendrocytes were identified by their characteristically cleared cytoplasm and contrast-enhanced nuclei. To assess general pathology sections were stained with hematoxylin and eosin.

### Ethics

The project was carried out in accordance with the ethical standards of the Human Ethics Review Committee of the Sydney Local Health District (Royal Prince Alfred Hospital Zone project X14-0029) with approval from the Coroner’s Office, Department of Forensic Medicine, Glebe, New South Wales and in accordance with the Declaration of Helsinki as revised in 2000. The institutional review board waived the need for written informed consent from relatives of the participant since this was a de-identified retrospective study of autopsy-obtained tissue.

## Results

### General histology

No abnormities were seen on hematoxylin and eosin sections of the cerebral cortex or white matter. In particular, there was no evidence of neuronal or oligodendrocyte cell loss, astrocytic hypertrophy (i.e., no visible eccentric perinuclear eosinophilic astrocytic cytoplasm) or destructive tissue damage leading to microglial activation (i.e., no foamy macrophages).

### Astrocyte architecture

**Fig. 1:**
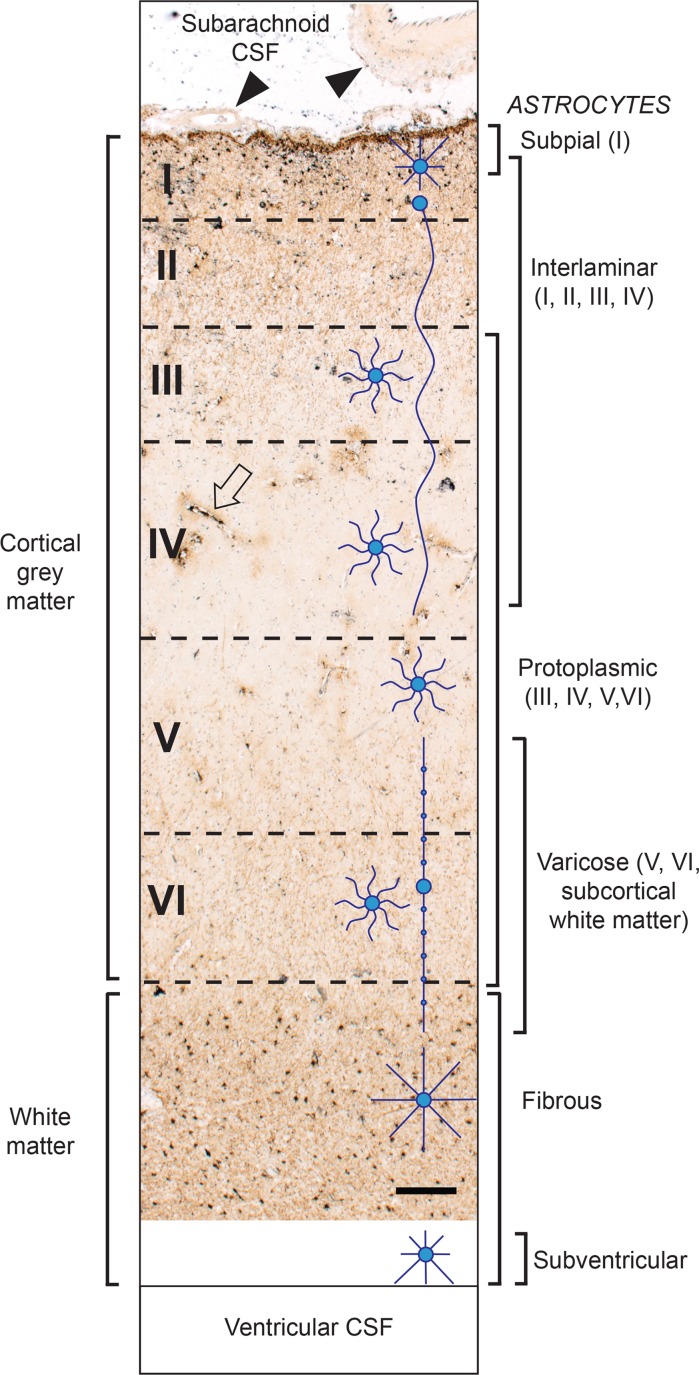
Astrocytes in the neocortical grey matter and subcortical white matter. Diagrammatic representations of astrocyte types are on the right (including the subventricular astrocytes not present in this section). Subpial astrocytes are found in layer I of the grey matter. Interlaminar astrocytes have their cell bodies in layer I, with long undulating processes extending to layers II, III and IV. Protoplasmic astrocytes are found in layers III–VI. Varicose astrocyte with their long beaded processes are found in layers V and VI with some processes extending into the subcortical white matter. Fibrous astrocytes are present in the white matter. A potential chain of interconnecting astrocytes stretches from the subpial to the subventricular region. Some intracerebral microvessels contain iHg (e.g., arrow; also see Fig. 4), but small (left) and large (right) subarachnoid blood vessels (arrowheads) are iHg-free. AMG^Hg^/GFAP/hematoxylin. Bar = 250 μm

GFAP immunostaining allowed visualisation of all astrocyte subtypes in the cortex and white matter (Fig. 1). The cell bodies of subpial and interlaminar astrocytes were in cortical layers I, protoplasmic astrocytes in layers III to VI, varicose astrocytes mostly in layer VI and fibrous astrocytes in the white matter. The long undulating processes of interlaminar astrocytes and the long beaded processes of varicose astrocytes were noted.

### Cortical grey matter

#### Subpial astrocytes

**Fig. 2:**
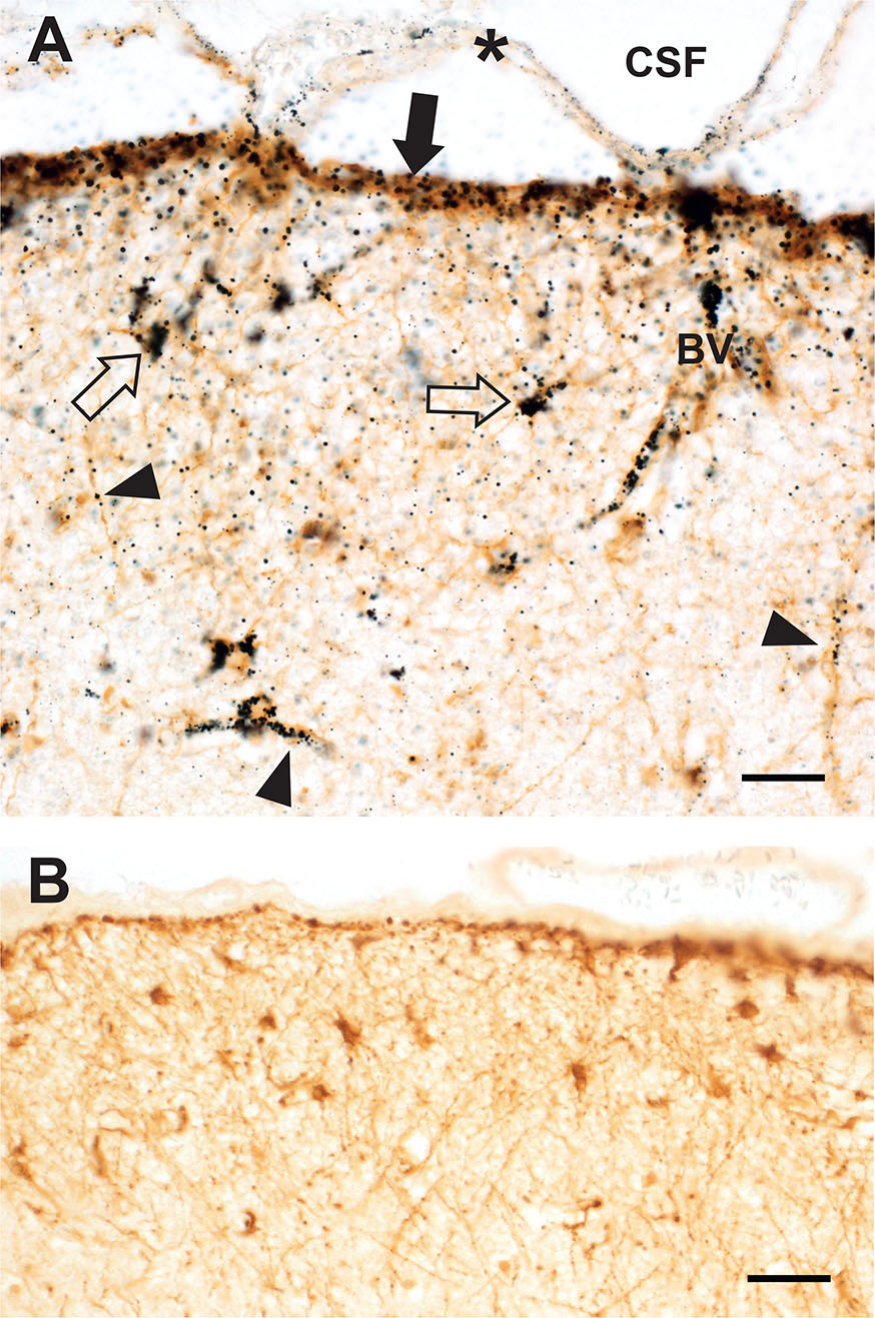
Inorganic mercury in grey matter subpial astrocytes. **a** The subpial glia limitans stains strongly for iHg (filled arrow). Cell bodies of subpial astrocytes in layer I contain iHg (e.g., open arrows). Small iHg grains can be seen in astrocytic processes (arrowheads) belonging to either subpial or interlaminar astrocytes. The wall of a penetrating blood vessel (BV) contains iHg. The pia mater (asterisk, artefactually separated from the underlying cortex) contains no significant iHg. AMG^Hg^/GFAP/hematoxylin. **b** A control section without AMG^Hg^ staining from the same region shows no black deposits within the brown subpial astrocyte cell bodies or processes. GFAP/hematoxylin. Bars = 20 μm

iHg was prominent in the glia limitans and the immediate subpial regions of cortical layer I where the cell bodies and processes of subpial astrocytes contained dense iHg (Fig. 2).

#### Interlaminar astrocytes

**Fig. 3:**
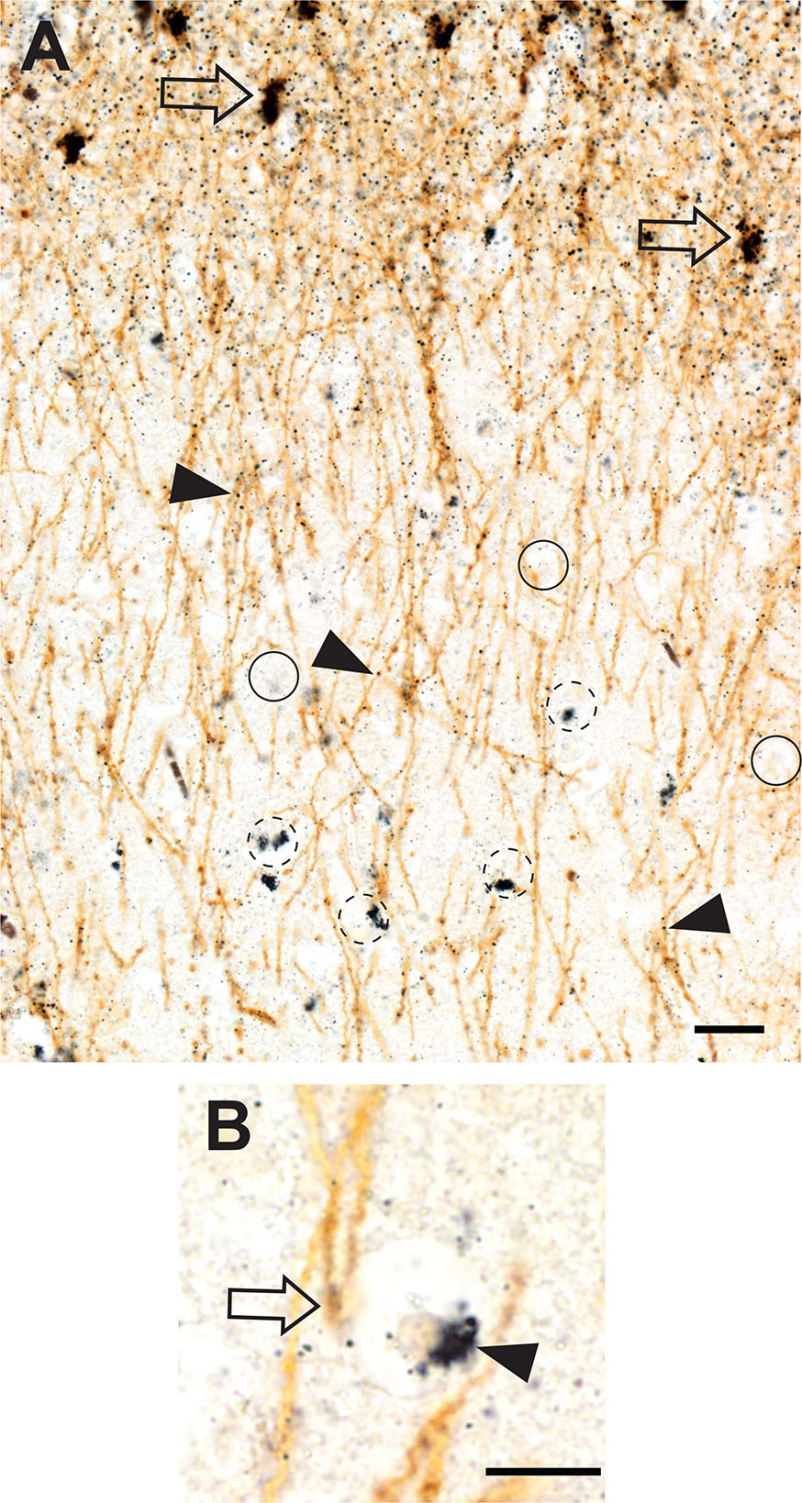
Inorganic mercury in grey matter interlaminar astrocytes and oligodendrocytes. **a** The cell bodies of interlaminar astrocytes in layer I (open arrows) stain strongly for iHg. iHg grains (arrowheads) are seen in their long undulating processes. A group of oligodendrocytes have iHg in their cytoplasm (e.g., dashed circles) whereas others are iHg-free (e.g., circles). Bar = 20 μm. **b** A high power view shows the cell body of one oligodendrocyte with a mostly empty white perikaryon. iHg has been concentrated into its artefactually shrunken cytoplasm (arrowhead) to the right of a pale-staining nucleus. The descending process of an interlaminar astrocyte appears to terminate in an end bulb (open arrow) on the surface of the oligodendrocyte. AMG^Hg^/GFAP/hematoxylin. Bar = 10 μm

The cell bodies of interlaminar astrocytes in the deeper parts of cortical layer I contained dense iHg (Fig. 3a). Individual iHg grains could be seen within the long undulating processes of the intralaminar astrocytes, some of which appeared to terminate on oligodendrocyte cell bodies in cortical layers II and III (Fig. 3b).

#### Protoplasmic astrocytes

**Fig. 4:**
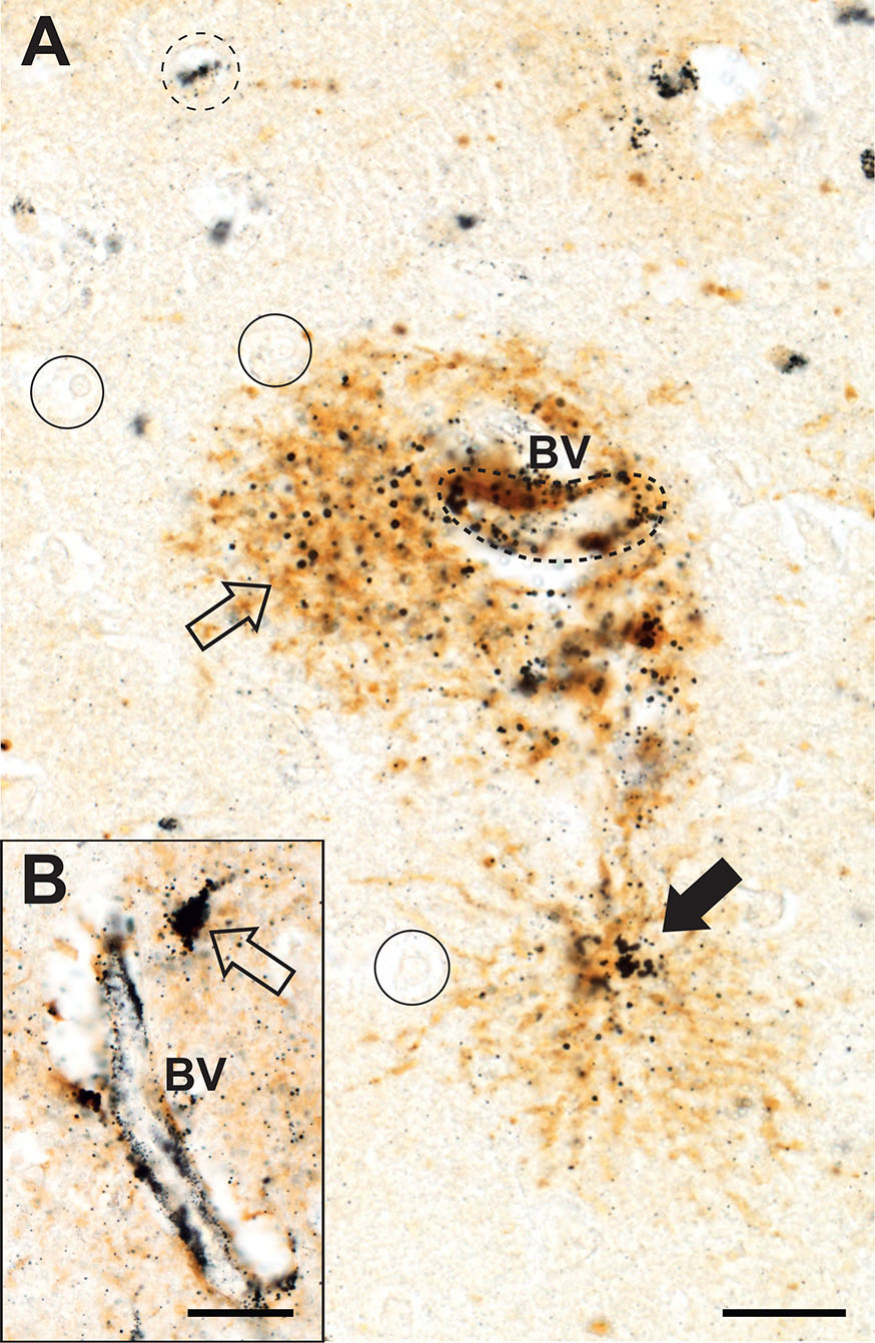
Inorganic mercury in grey matter protoplasmic astrocytes, oligodendrocytes and microvessels. **a** The wall of a blood vessel (BV, dashed outline) in layer III stains for iHg. An adjacent protoplasmic astrocyte (open arrow) contains numerous iHg grains as does a second astrocyte (closed arrow) a short distance from the microvessel. One oligodendrocyte (dashed circle) contains iHg but most oligodendrocytes (e.g., circles) are iHg-free. **b** The wall of a small blood vessel (BV) stains strongly for iHg as does the cell body of an adjacent protoplasmic astrocyte (open arrow). AMG^Hg^/GFAP/hematoxylin. Bars = 20 μm

The cell bodies of protoplasmic astrocytes, usually adjacent to microvessels, often contained dense iHg, and their processes had numerous individual iHg grains (Fig. 4a, b). Protoplasmic astrocytes immediately adjacent to the perivascular astrocytes also contained iHg. Cortical layers III and IV had the greatest concentration of iHg-containing protoplasmic astrocytes.

#### Varicose astrocytes

**Fig. 5:**
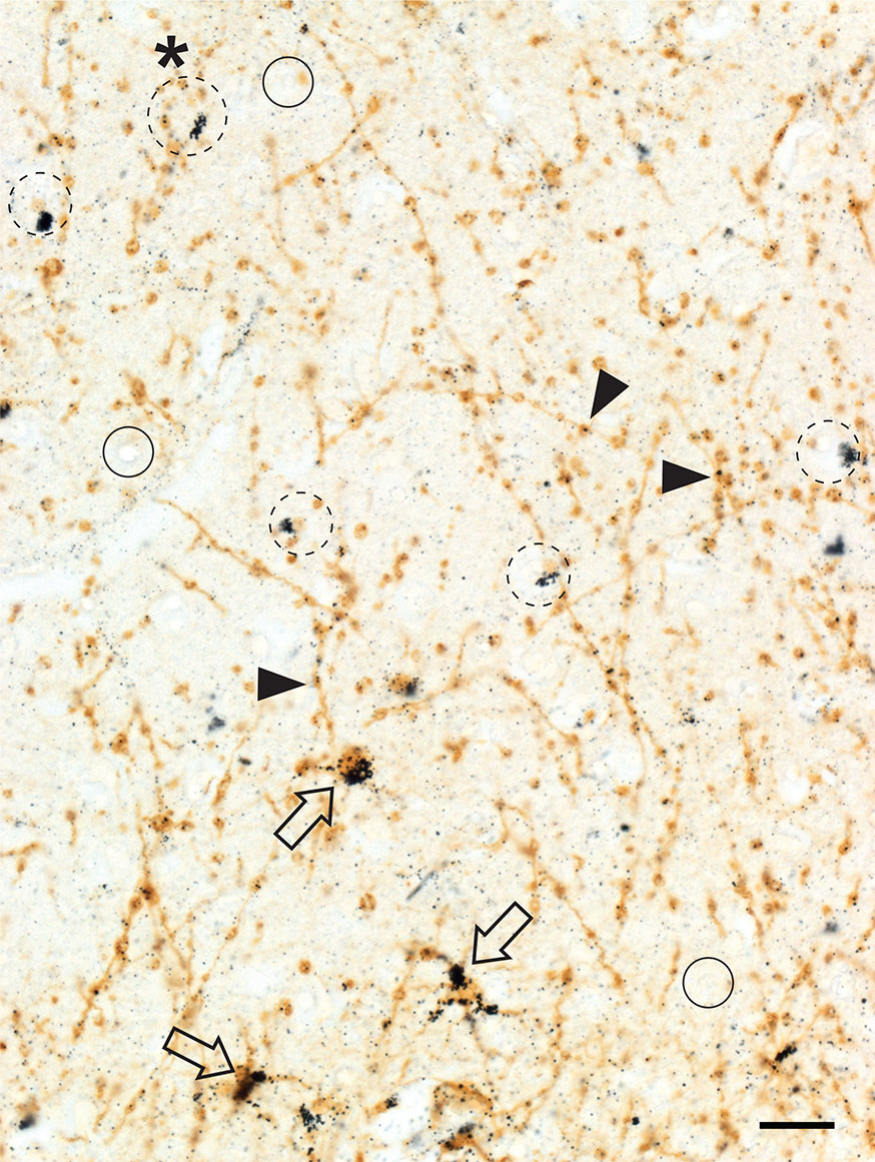
Inorganic mercury in grey matter varicose astrocytes and oligodendrocytes. Cell bodies of three varicose astrocytes (open arrows) in layer VI contain iHg. Many of the varicosities on these straight processes contain an iHg grain (arrowheads). Some oligodendrocytes (e.g., dashed circles) contain iHg though others are iHg-free (e.g., circles). Varicose astrocyte processes pass close to many iHg-containing oligodendrocytes (e.g., in the dashed circle with an asterisk). AMG^Hg^/GFAP/hematoxylin. Bar = 20 μm

The cell bodies of varicose astrocytes in cortical layers V and VI contained large amounts of iHg (Fig. 5). The varicosities on the processes of these astrocytes appeared to have a predilection for iHg, with only a few grains in the processes between the varicosities. Varicose astrocytic cell processes were often seen in close proximity to oligodendrocytes.

#### Oligodendrocytes

Grey matter iHg-containing oligodendrocytes were seen in greatest numbers in cortical layers II and III (Fig. 3a), with fewer in layers IV, V and VI and none in layer I. These iHg-containing oligodendrocytes did not appear uniformly along the cortical ribbon but rather in irregularly-separated loose clusters. In paraffin sections oligodendrocyte cell body cytoplasm was artefactually shrunken due to tissue processing, leaving most of perikaryon as an empty space. iHg in oligodendrocyte cytoplasm was consequently visible as a dense cluster of AMG^Hg^ staining occupying a small area of the shrunken cytoplasm, usually adjacent to the nucleus (Fig. 3b).

#### Corticomotoneurons

**Fig. 6:**
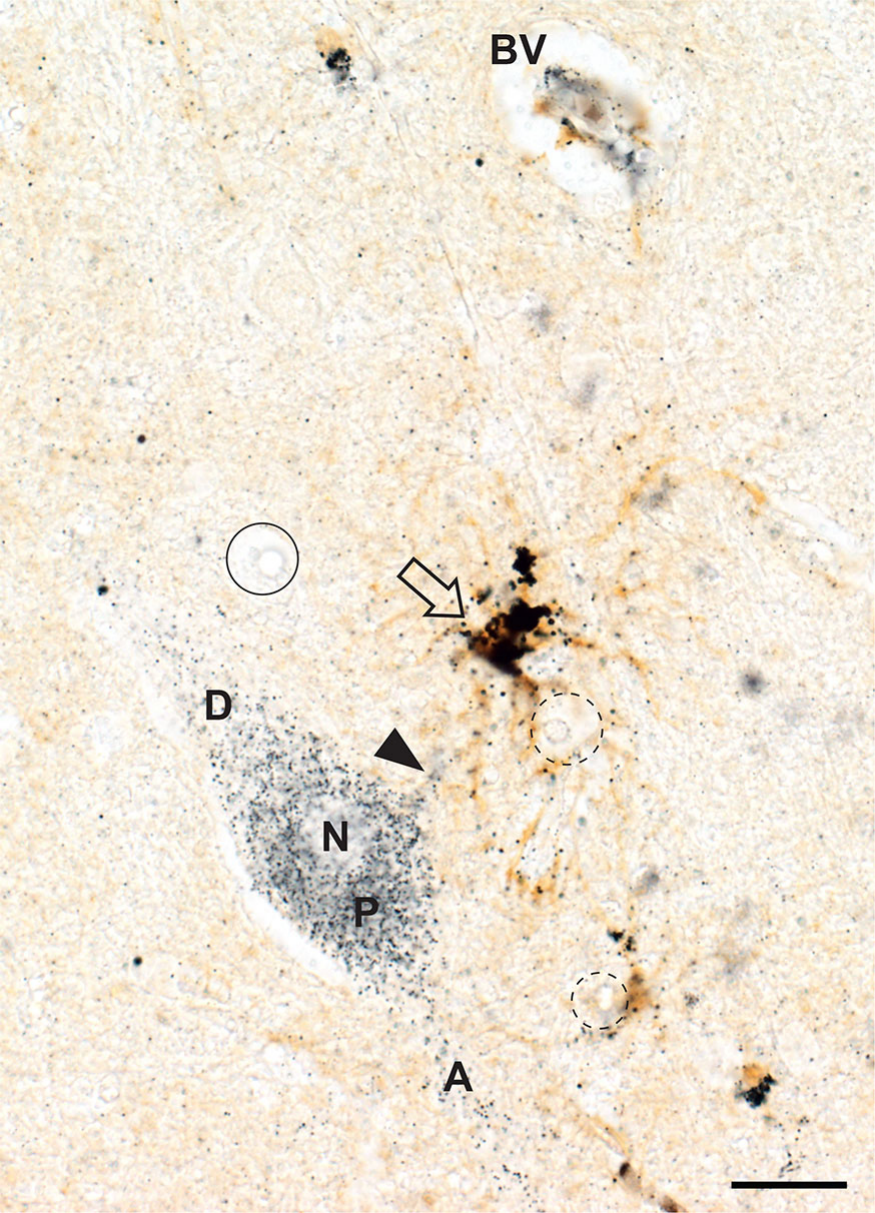
Inorganic mercury in a corticomotoneuron and adjacent astrocyte. In layer V of the primary motor cortex a corticomotoneuronal perikaryon (P), axon (A) and dendrite (D) contain numerous iHg grains (N = nucleus). The cell body and processes of a nearby protoplasmic astrocyte (arrow) stain strongly for iHg, possibly from iHg taken by from a nearby blood vessel (BV). A small dendrite (arrowhead) of the corticomotoneuron makes close contact with an astrocytic process. Oligodendrocytes do not contain iHg (e.g., circle) despite some (dashed circles) being in close contact with iHg-bearing astrocytic processes. AMG^Hg^/GFAP/hematoxylin. Bar = 20 μm

All corticomotoneurons in cortical layer 5 of the primary motor cortex had dense iHg in their cell bodies and neurites (Fig. 6). A heavily iHg-stained protoplasmic astrocyte could often be seen interposed between a corticomotoneuron and a nearby microvessel (Fig. 6). No other neurons in either the motor or sensory cortex contained iHg.

#### Blood vessels

Large and small extracerebral blood vessels in the subarachnoid space overlying the cortex contained no significant iHg (Fig. 1). In the cortex a number of thin-walled post-capillary venules (i.e., with diameters above 20 μm) had iHg grains in their walls (Fig. 1). These iHg-stained post-capillary venules appeared most prominently in cortical layers III and IV.

### White matter

#### Fibrous astrocytes

**Fig. 7:**
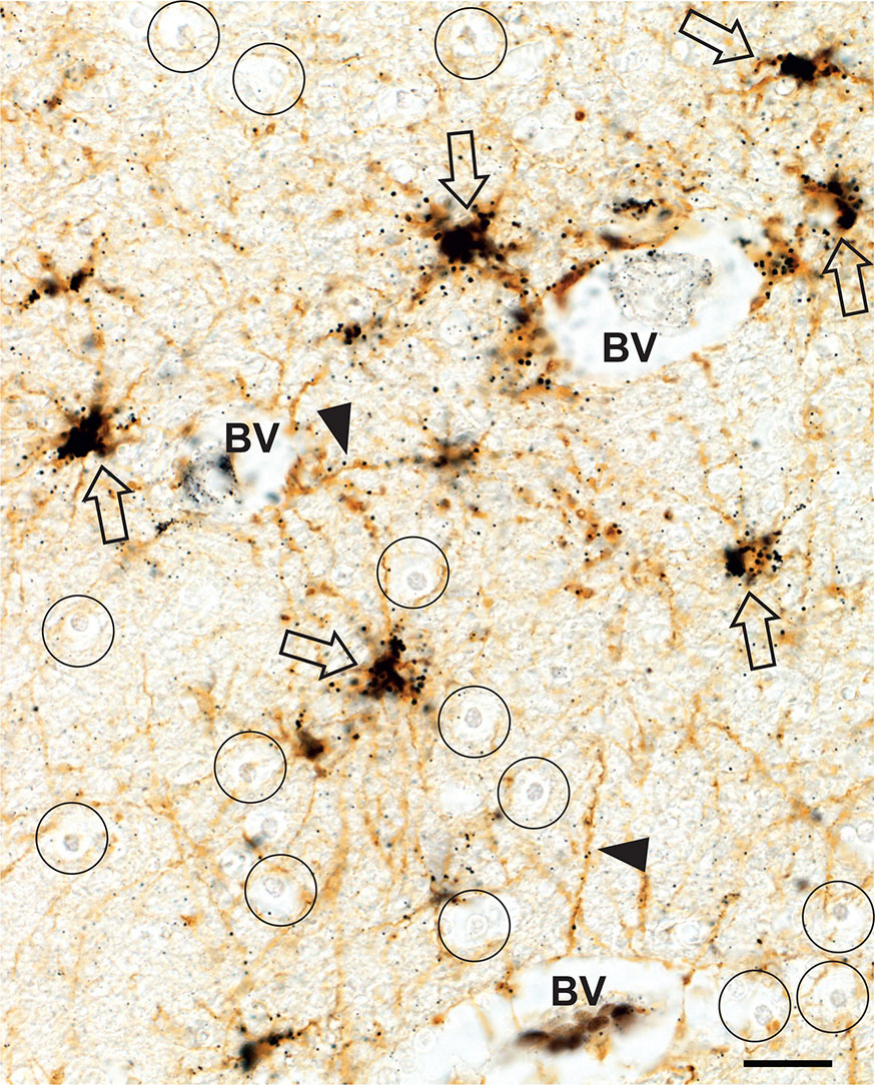
Inorganic mercury in white matter fibrous astrocytes and microvessels. The cell bodies of all fibrous astrocytes (open arrows) contain abundant iHg. Many astrocytic processes contain iHg grains (arrowheads). Some astrocytic processes terminate at the glia limitans of artefactually expanded perivascular spaces around blood vessels (BV). None of the numerous white matter oligodendrocytes (e.g., circles) contain iHg. AMG^Hg^/GFAP/hematoxylin. Bar = 20 μm

All fibrous astrocytes in the white matter had dense iHg in their cytoplasm and numerous iHg grains in their processes (Fig. 7). The processes of perivascular fibrous astrocytes could often be seen to extend to the edge of the nearby perivascular spaces. Fibrous astrocytes with no visible processes connecting with microvessels also contained dense iHg.

#### Oligodendrocytes

None of the many oligodendrocytes in the white matter contained iHg (Fig. 7).

#### Blood vessels

A number of white matter post-capillary venules contained iHg grains in their walls, with varying degrees of staining between the different venules (Fig. 7).

### Locus ceruleus

**Fig. 8:**
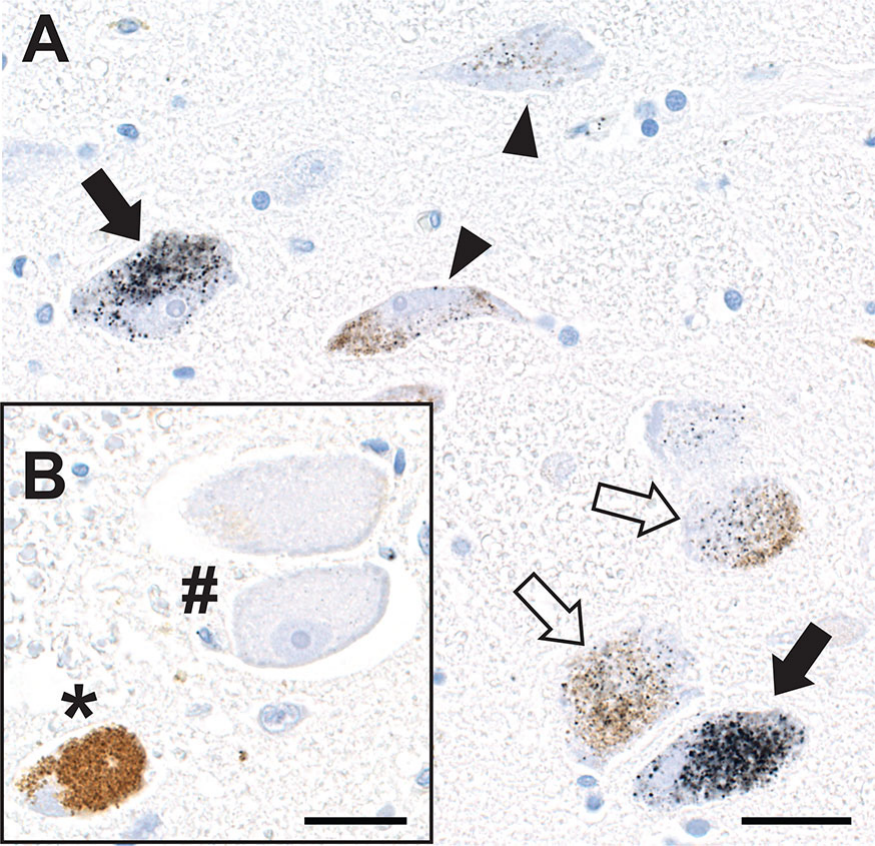
Inorganic mercury in locus ceruleus neurons. **a** The cytoplasm of locus ceruleus neurons contains either dense (closed arrows), light (open arrows) or insignificant (arrowheads) amounts of iHg. **b** At the margin of the locus ceruleus two large neurons (hash) of the trigeminal mesencephalic nucleus contain no iHg. An adjacent neuromelanin-containing locus ceruleus neuron (asterisk) also contains no iHg. AMG^Hg^/hematoxylin. Bar = 20 μm

Locus ceruleus neurons showed iHg staining which varied from heavy to none, without any regular pattern of staining (such as being close to a blood vessel) so that a densely-stained neuron could be seen with a juxtaposed non-stained neuron (Fig. 8a). None of the other neurons in the pons, including the trigeminal mesencephalic neurons bordering the locus ceruleus, contained iHg (Fig. 8b).

## Discussion

In a man who injected himself with metallic mercury iHg was detected in all five major types of human astrocytes, scattered grey matter oligodendrocytes, corticomotoneurons, locus ceruleus neurons and cerebral microvessels. The glial and microvessel findings were similar in the primary motor and sensory cortices. iHg could be seen in protoplasmic and fibrous astrocytes, both adjacent to and at some distance from microvessels, suggesting iHg was transferred from perivascular astrocytes to adjacent astrocytes, possibly via the gap junctions that connect these cells (Orthmann-Murphy et al. 2008). The long processes of interlaminar and varicose also contained iHg, raising the possibility that iHg can be transferred along a chain of astrocytes between the subpial cortex and the subcortical white matter. The microvessels with iHg in or around their walls had the features of post-capillary venules, the most permeable of the brain blood vessels (Holman et al. 2011). Only grey matter oligodendrocytes contained iHg, suggesting that astrocyte-oligodendrocyte gap junctions in either cortical protoplasmic, interlaminar or varicose astrocytes transferred iHg to these oligodendrocytes (Orthmann-Murphy et al. 2008). Protoplasmic astrocytes adjacent to iHg-containing corticomotoneurons seemed particularly prone to accumulate iHg. Dense collections of iHg immediately below the pia mater suggests that iHg from the subarachnoid cerebrospinal fluid permeated into the superficial cortex where it was taken up by subpial astrocytes. Finally, scattered locus ceruleus neurons contained large amounts of iHg; since noradrenaline from the locus ceruleus plays a major role in maintaining the blood–brain barrier (Harik and McGunigal 1984) any diminished noradrenaline output due to iHg toxicity in these scattered locus ceruleus neurons could make the blood–brain barrier more permeable to circulating iHg in focal regions of the brain.

Astrocytes are known to selectively accumulate circulating mercury (Tiffany-Castiglion and Qian 2001). Primate studies indicate that after mercury exposure iHg localises first to astrocytes and then months later, and to a lesser extent, in neurons, followed by either losses or proliferation of astrocytes (Charleston et al. 1996). Of interest was the finding that all five major types of human astrocytes contained iHg deposits in their cell bodies and processes. Two of these, the interlaminar and varicose astrocytes, are present only in higher primates (Oberheim et al. 2006). Although we did not examine the subventricular region, the astrocytes here are also found predominantly in humans (Sanai et al. 2004). The discovery of a neurotoxin in primate-specific astrocytes is of possible relevance to the pathogenesis of diseases that occur mostly in humans, such as multiple sclerosis, Alzheimer’s disease and ALS and could explain why rodent models of these diseases seldom reproduce comprehensive features of the human disorders (Lassmann and Bradl 2017). Finally, finding a neurotoxicant in the long processes of two primate-specific astrocytes, together with the existence of gap junctions between astrocytes, implies that long astrocytic chains may exist in the human brain (Bazargani and Attwell 2016) which are potential pathways for toxicants or misfolded proteins to spread within the CNS (Cushman et al. 2010).

**Fig. 9:**
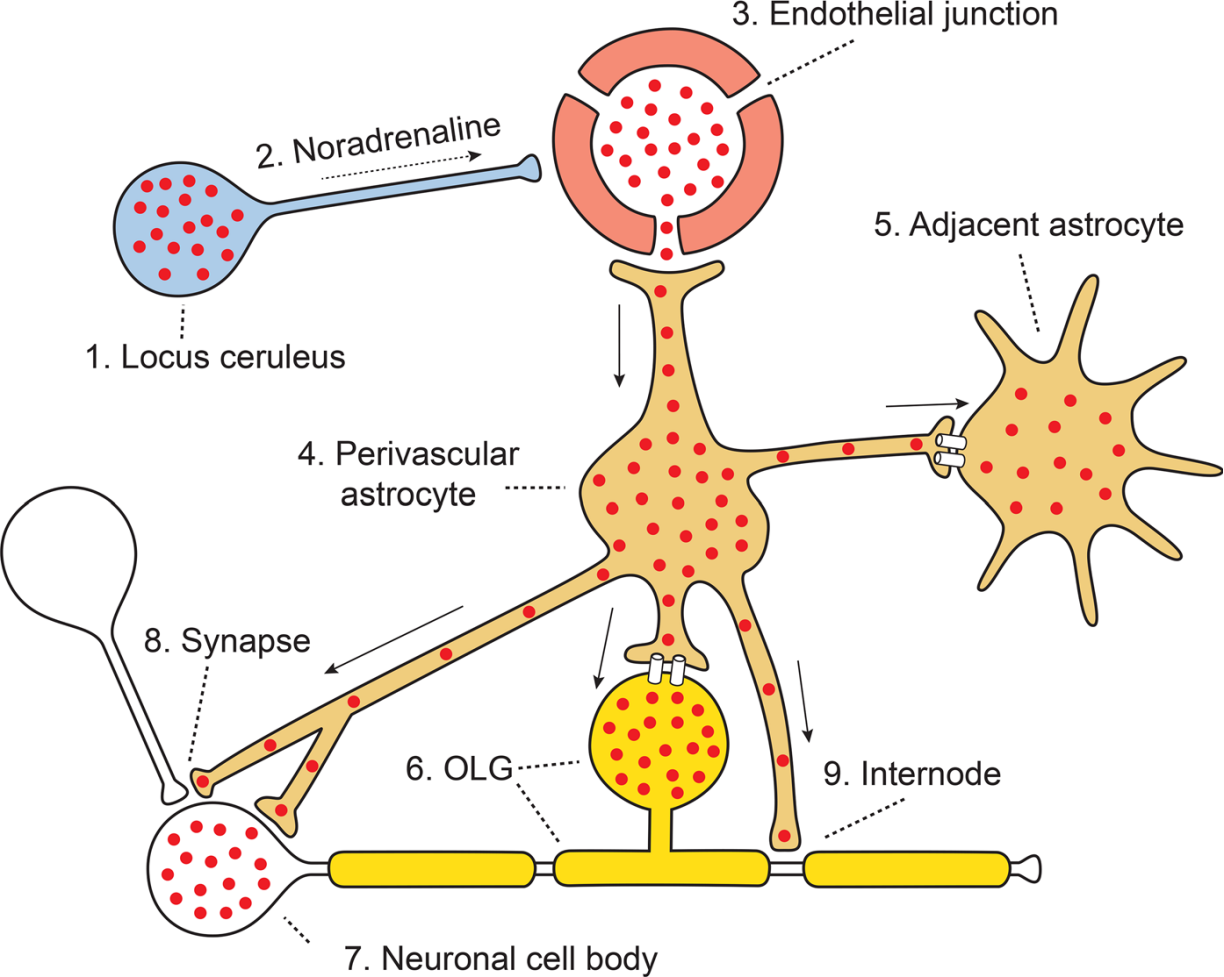
Model of pathways inorganic mercury uses to enter the human brain. iHg (red dots) is taken up selectively by a locus ceruleus neuron (1) and decreases noradrenaline output (2) to cerebral microvessels supplied by this neuron. This increases endothelial permeability to circulating iHg (3). Perivascular astrocytes (4) take up the iHg and transfer it via intercellular gap junctions to adjacent astrocytes (5) and to grey matter oligodendrocytes (OLG) (6). iHg-containing astrocytes damage nearby neurons via metabolic disturbances in neuronal cell bodies (7), synapses (8) and myelin internodes (9). Astrocytes directly transfer iHg into the corticomotoneuronal cytoplasm (7)

Our findings have led us to propose a model for routes of entry of iHg into the brain which may have pathogenetic implications for a range of neurological disorders (Fig. 9). In this model, the locus ceruleus selectively take up iHg, after which reduced noradrenaline output increases blood–brain barrier permeability, encouraging the passage of iHg through cerebral microvessels into the perivascular space. Perivascular astrocytes then take up the iHg and pass it on to adjacent astrocytes and grey matter oligodendrocytes via gap junctions, with subsequent damage to these oligodendrocytes. White matter oligodendrocytes on the other hand are damaged from iHg binding to gap junctions (Piccoli et al. 2012) thereby inhibiting transfer of nutrients from astrocytes to these oligodendrocytes. Damage to neurons results from a combination of the deleterious effects of iHg on astrocyte support to neurons, synapses (Haydon 2001) and internodes, as well as iHg damage to oligodendrocytes. In corticomotoneurons the direct transfer of iHg from astrocytes damages these neurons.

The potential significance of finding iHg in human brain cells becomes evident when consideration is given to the subcellular locations of mercury. Gap junctions are affected by iHg at low levels (Piccoli et al. 2012) which could disrupt communication between astrocytes and oligodendrocytes. Mitochondria are implicated in a number of neurodegenerative diseases and iHg localises to mitochondria (Chang and Hartmann 1972). Mercury has a particular affinity for the sulfhydryl groups of cysteine-containing proteins (Clarkson and Magos 2006) found in lipid membranes of the Golgi apparatus, the endoplasmic reticulum and lysosomes, all of which have been linked to neurodegenerative diseases (Farina and Aschner 2017). Furthermore, ultrastructural studies have shown iHg binds to all these lipid-rich sites in the nervous system (Chang and Hartmann 1972).

**Fig. 10:**
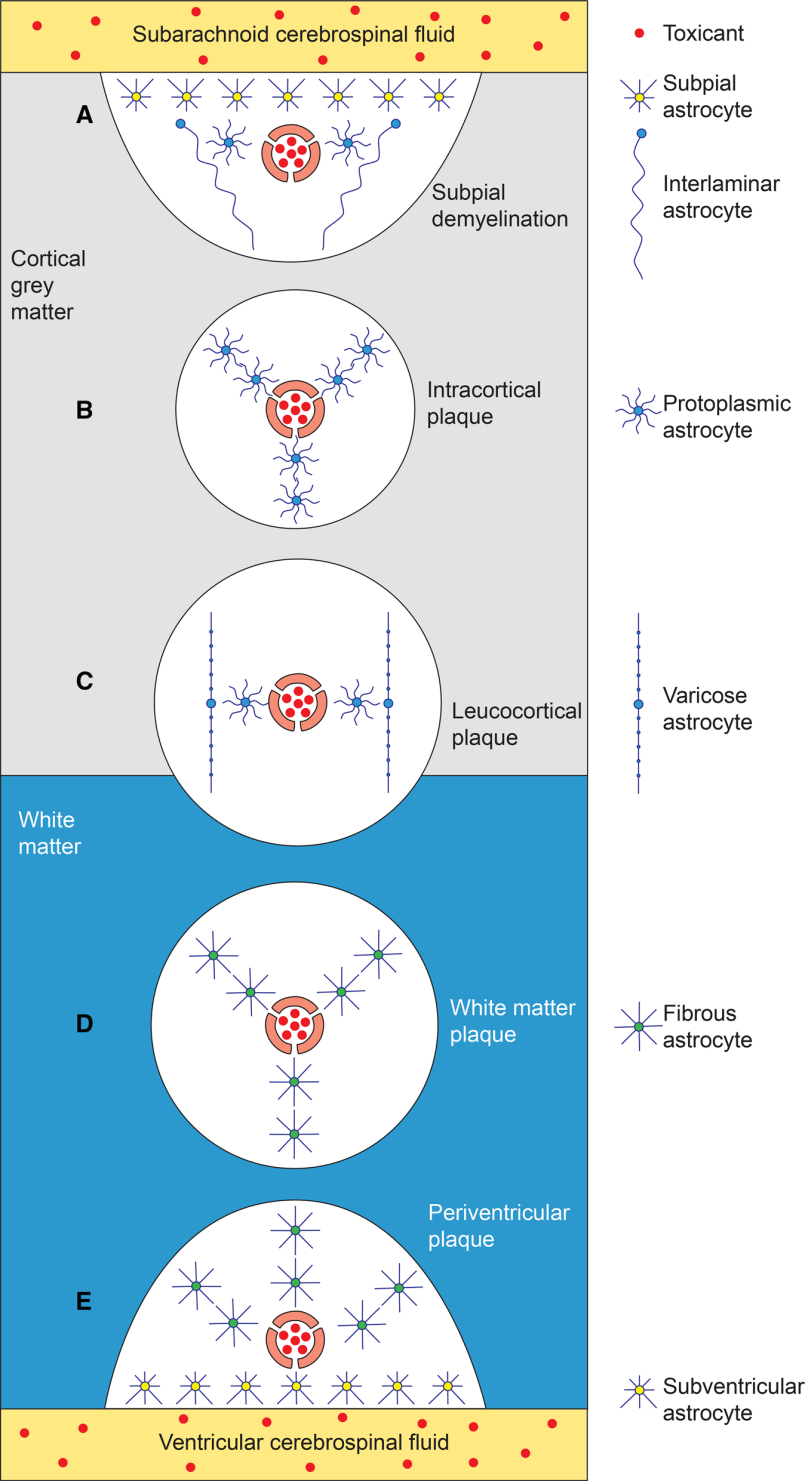
Model of demyelination in multiple sclerosis in relation to the anatomical distribution of astrocyte subtypes. A toxicant (such as iHg, red dots) is present in small intracerebral blood vessels; the blood–brain-barrier of these microvessels has been impaired by a noradrenaline deficit due to iHg-damaged locus ceruleus neurons. The cerebrospinal fluid in both the subarachnoid space and ventricles contains iHg. Perivascular and cerebrospinal fluid iHg then spreads via communicating networks of astrocytes to give rise to the five anatomical patterns of demyelination in multiple sclerosis: **a** *Subpial demyelination* arises from iHg uptake from the subarachnoid cerebrospinal fluid by subpial astrocytes and from microvessels by interconnected protoplasmic and interlaminar astrocytes. **b** Small *intracortical plaques* arise from microvessel iHg uptake by interconnected protoplasmic astrocytes. **c** *Leucocortical plaques* arise from uptake of microvessel iHg by protoplasmic and interconnected varicose astrocytes. **d** *Deep white matter plaques* originate from microvessel iHg uptake by interconnected fibrous astrocytes. **e** *Periventricular white matter plaques* arise from a combination of iHg uptake by subventricular astrocytes from ventricular cerebrospinal fluid and from microvessels by interconnected fibrous astrocytes

In multiple sclerosis a primary role for astrocytes has been postulated (Brosnan and Raine 2013) and astrocytes proliferate markedly in otherwise normal multiple sclerosis white matter (Allen 1981). The demyelination in neuromyelitis optica, a disorder related to multiple sclerosis, results from an autoimmune attack on aquaporin-4 in astrocytic foot processes (Lucchinetti et al. 2014); this may be relevant to other demyelinating conditions that could be triggered by mercury, since aquaporins are inhibited by mercury (Ximenes-da-Silva 2016). iHg-induced damage to astrocytes could augment the neuroinflammation seen in multiple sclerosis since astrocytes are key regulators of brain immune responses (Colombo and Farina 2016). The characteristic patterns of demyelination in multiple sclerosis, i.e., subpial, intracortical, leucocortical, deep white matter and periventricular white matter (Mahad et al. 2015) may be linked to the spread of iHg in different astrocytic pathways within the human brain; such a model is illustrated in Fig. 10. The white matter oligodendrocyte loss and subsequent demyelination in multiple sclerosis could be secondary to astrocytic damage from iHg, and the progressive grey matter damage (Mahad et al. 2015) due to iHg within cortical oligodendrocytes. Furthermore, gap junction or mitochondrial damage from iHg may be responsible for the “virtual hypoxia” of myelin suggested to be responsible for the lesions of multiple sclerosis (Lassmann 2018). Finally, the locus ceruleus has been implicated in multiple sclerosis because of its role in maintaining the blood–brain barrier and because of its ability to suppress neuroinflammation (Feinstein et al. 2016). Therefore a number of lines of evidence suggest a neurotoxin such as iHg could play a role in the pathogenesis of multiple sclerosis.

Astrocyte pathology has been suggested to be an important feature in Alzheimer’s disease (Pekny et al. 2016): astrocytes are associated with Alzheimer neuritic plaques (Cullen 1997), interlaminar astrocytes are specifically lost in the disease (Colombo et al. 2002), astrocytes accumulate abnormally phosphorylated tau protein (Kovacs et al. 2017) and mercury has been shown to hyperphosphorylate tau (Fujimura et al. 2009). Oligodendrocyte damage too has been described in Alzheimer’s disease (Cai and Xiao 2016) and the locus ceruleus is one of the earliest sites of Alzheimer pathology (Braak and Del Tredici 2011). Our findings of iHg in astrocytes, oligodendrocytes and the locus ceruleus therefore suggest more investigations of heavy metals as a trigger for Alzheimer’s disease are warranted.

Mercury has been suspected to be involved in the pathogenesis of ALS (Andrew et al. 2018). A striking feature in the frontal motor cortex in our study was the presence of iHg in corticomotoneurons, whereas no other motor or sensory cortical neurons contained iHg. iHg was also present in nearby astrocytes, raising the possibility that iHg could be transferred directly from these astrocytes into the corticomotoneuron cell body, perhaps via extracellular vesicles or some other form of intercellular connection (Bellingham et al. 2015) since gap junctions do not appear to be present between adult astrocytes and neurons (Rash et al. 2001). On the other hand, it is possible that iHg is moving in the opposite direction, from corticomotoneuron to astrocyte, in an attempt to remove mercury from the neuron (Tiffany-Castiglion and Qian 2001). ALS is suspected to originate in corticomotoneurons (Eisen and Weber 2001) so it is of interest that mercury inhibits astrocytic glutamate uptake, leaving more of this excitatory amino acid in the synaptic cleft to damage the neuron (Shanker et al. 2003). The finding of iHg in corticomotoneurons and in other neurons implicated in ALS (Pamphlett and Kum Jew 2016) raises the possibility that iHg exerts its neurotoxic effect by binding to cysteine that forms the intra-superoxide dismutase 1 disulphide bonds, thereby preventing its normal folding and leading to misfolded forms of the protein (Sea et al. 2015); this has been found in the spinal cord in both familial and sporadic ALS (Gruzman et al. 2007) and by this mechanism iHg could produce a neurotoxic phenocopy of superoxide dismutase 1-mutant ALS. Numerous other functions of astrocytes have been suggested to be disturbed in ALS (Yamanaka and Komine 2018) and motor neuron death can be triggered by astrocytes (Re et al. 2014). Oligodendrocyte too appear to play a role in ALS (Philips et al. 2013). Therefore the presence of iHg in astrocytes, oligodendrocytes and corticomotoneurons may have relevance to the pathogenesis of ALS.

Brain cells appear to be particularly sensitive to the genotoxic effects of mercury (Crespo-Lopez et al. 2009) and mercury can cause epigenetic modifications (Ray et al. 2014) which together with DNA mutations may be responsible for the development of gliomas (Caffo et al. 2014). The possibility that mercury could be a trigger for glioblastomas, the most malignant form of astrocytoma, was initially raised because of a report that dentists and dental nurses had an increased risk for these tumours (Ahlbom et al. 1986), though these results were not repeated in other studies (Boffetta et al. 1993). Oligodendrogliomas have been reported to be common in the cortical-subcortical region of the brain (Smits 2016), a region in our study where cortical oligodendrocytes often contained iHg. So it is of interest that these two common primary brain tumours, glioblastoma and oligodendroglioma, arise from astrocytes and oligodendrocytes, which contain large amounts of potentially-mutagenic iHg after human exposure to mercury.

In conclusion, in a man who injected himself with metallic mercury we found inorganic mercury in cells that are involved in the pathogenesis of multiple sclerosis, Alzheimer’s disease, ALS and brain tumours. Many of the pathophysiological mechanisms thought to underlie these disorders are mirrored by the multiple deleterious effects of intracellular mercury. Elemental analysis studies of mercury and other neurotoxicants within the cells of the human brain are warranted to further investigate the contributions of neurotoxins to this range of disorders.
